# Bioactive Chemical Constituents from the Brown Alga *Homoeostrichus formosana*

**DOI:** 10.3390/ijms16010736

**Published:** 2014-12-30

**Authors:** Hui-Yu Fang, Uvarani Chokkalingam, Shu-Fen Chiou, Tsong-Long Hwang, Shu-Li Chen, Wei-Lung Wang, Jyh-Horng Sheu

**Affiliations:** 1 Department of Marine Biotechnology and Resources, National Sun Yat-sen University, Kaohsiung 80424, Taiwan; E-Mails: ahui0220@yahoo.com.tw (H.-Y.F.); uvaranichem@gmail.com (U.C.); sfc@mail.nsysu.edu.tw (S.-F.C.); 2 Graduate Institute of Natural Products, Chang Gung University, Taoyuan 33302, Taiwan; E-Mail: htl@mail.cgu.edu.tw; 3 Chinese Herbal Medicine Research Team, Healthy Aging Research Center, Chang Gung University, Taoyuan 33302, Taiwan; 4 Graduate Institute of Natural Products, Kaohsiung Medical University, Kaohsiung 80708, Taiwan; E-Mail: slchen@kmu.edu.tw; 5 Department of Biology, National Changhua University of Education, Changhua 50007, Taiwan; E-Mail: wlwang@cc.ncue.edu.tw; 6 Department of Medical Research, China Medical University Hospital, China Medical University, Taichung 40402, Taiwan

**Keywords:** *Homoeostrichus formosana*, chromenes, cytotoxicity, antibacterial, anti-inflammatory

## Abstract

A new chromene derivative, 2-(4',8'-dimethylnona-3'*E*,7'-dienyl)-8-hydroxy-2,6-dimethyl-2*H*-chromene (**1**) together with four known natural products, methylfarnesylquinone (**2**), isololiolide (**3**), pheophytin a (**4**), and β-carotene (**5**) were isolated from the brown alga *Homoeostrichus formosana*. The structure of **1** was determined by extensive 1D and 2D spectroscopic analyses. Acetylation of **1** yielded the monoacetylated derivative 2-(4',8'-dimethylnona-3'*E*,7'-dienyl)-8-acetyl-2,6-dimethyl-2*H*-chromene (**6**). Compounds **1**–**6** exhibited various levels of cytotoxic, antibacterial, and anti-inflammatory activities. Compound **2** was found to display potent *in vitro* anti-inflammatory activity by inhibiting the generation of superoxide anion (IC_50_ 0.22 ± 0.03 μg/mL) and elastase release (IC_50_ 0.48 ± 0.11 μg/mL) in FMLP/CB-induced human neutrophils.

## 1. Introduction

**Chart 1:**
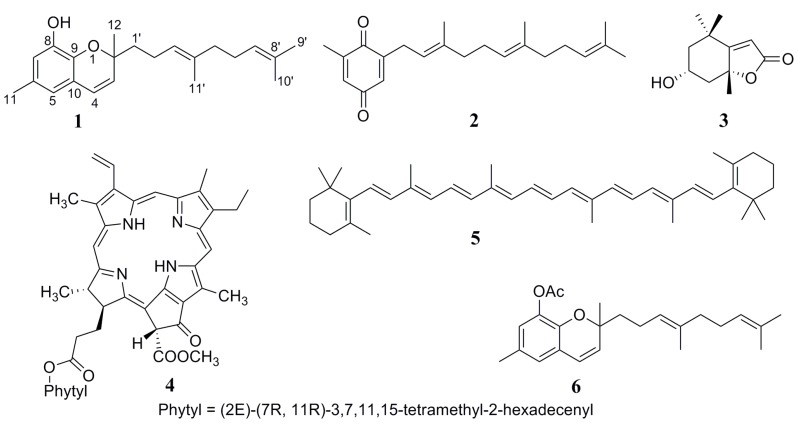
Structures of metabolites **1**–**6**.

In continuation of our search for bioactive chemical constituents from marine alga [1,2,3,4,5,6], we found that a crude acetone extract of *Homoeostrichus formosana* (Dictyotaceae, Phaeophyceae) [7] displayed significant cytotoxic and anti-inflammatory properties. A novel chromene 2-(4',8'-dimethylnona-3'*E*,7'-dienyl)-8-hydroxy-2,6-dimethyl-2*H*-chromene (**1**), along with four known compounds methylfarnesylquinone (**2**) [8], isololiolide (**3**) [9], pheophytin a (**4**) [10,11], and β-carotene (**5**) [12] (Chart 1) were identified for the first time using a bioassay-directed fractionation method. Meroterpenoids of the chromene skeleton and related structural units incorporated with a polyprenyl chain, are particularly abundant in brown alga (division Phaeophyta), which makes chromenes one of the representative groups of secondary metabolites of these organisms [13,14,15]. 2-Substituted 2*H*-chromenes and their analogs are pervasive molecular structures in chemistry, biology, and medicine. Many of these compounds possess various interesting biological properties that includes antidiabetic [16], cytotoxic [17], antifungal [18], anti-HIV [19], antibacterial [20], antitumor [21] and potent antioxidant (vitamin E) [22,23] activities. Recently, we also reported cytotoxic, anti-inflammatory, and antibacterial properties of sulfur-containing polybromoindoles from the Formosan red alga *Laurencia brongniartii* [24]. Consequently, the study has been undertaken to assess the cytotoxic, antibacterial, and anti-inflammatory activities of isolated compounds **1**–**6**. Compounds **1**, **2**, and **4**–**6** were tested for cytotoxicity against HepG2, A-549, and MDA-MB-231 cancer cell lines as well as antibacterial activity against *Bacillus cereus*, *Staphylococcus aureus*, *Salmonella typhimurium*, *Pseudomonas aeruginosa*, *Serratia marcescens* and *Yersinia entercocolitica*. The abilities of **1**, **2**, **5**, and **6** to inhibit superoxide anion generation and elastase release in FMLP/CB induced human neutrophils were also studied. Herein, we describe the isolation and structure elucidation of compound **1**, and the results of biological activity assays of compounds **1**–**6**.

## 2. Results and Discussion

Compound **1** was obtained as a brown oil and its molecular formula was assigned as C_22_H_30_O_2_ by HRESIMS (High Resolution Electron Spray Ionization Mass Spectrometry) showing a [M + Na]^+^ peak at *m*/*z* 349.2143, implying eight degrees of unsaturation. The UV spectrum showed absorption maxima at 206, 231, 265, 274 and 333 nm, suggesting a conjugated aromatic ring system [25] and IR absorption bands indicated the presence of hydroxy (3396 cm^−1^), and an aromatic chromophore (1593, 1463 cm^−1^) [26]. The ^13^C-NMR spectrum (Figure S1) comprises resonances of 22 carbon signals and these signals were assigned to five methyls, four sp^3^ methylenes, six sp^2^ methines, six sp^2^ quaternary carbons and one sp^3^ quaternary carbon using a combination of DEPT (Distortionless Enhancement by Polarization Transfer) and HSQC (Heteronuclear Single Quantum Coherence) data (Figure S2 and Figure S3). The characteristic 2*H*-chromene moiety was inferred by the aromatic proton resonances (δ 6.34, 1H, d, *J* = 2.8 Hz; 6.49, 1H, d, *J* = 2.8 Hz), and AB type doublets (δ 5.60, 1H, d, *J* = 9.6 Hz; 6.25, 1H, d, *J* = 9.6 Hz) in the ^1^H-NMR spectrum (Figure S4), as well as eight sp^2^ carbons (δ 110.3 to 148.5) and one quaternary carbon at δ 77.8 in the ^13^C-NMR spectrum. The ^1^H-NMR spectrum indicated the presence of a geranylmethyl moiety, as evidenced by the resonances at δ 1.72 (2H, m), 2.12 (2H, m), 1.98 (2H, m), 2.06 (2H, m), 5.11 to 5.14 (2H, overlapped) and three methyl singlets. Further, the constitution of this side chain was confirmed by ^1^H–^1^H COSY (^1^H–^1^H correlation spectroscopy) (Figure S5) correlations from H_2_-1' to H_2_-2', H_2_-2' to H-3', H_2_-5' to H_2_-6' and H_2_-6' to H-7'. The HMBC (Heteronuclear Multiple Bond Correlation) (Figure S6) spectrum exhibited interactions of the H_3_-12 (δ 1.38, CH_3_) with C-2 (δ 77.8), C-3 (δ 130.7) and C-1' (δ 40.7), and the H-1' (δ 1.72) with C-2 (δ 77.8) and C-2' (δ 22.5). These HMBC data suggested attachment of the C-1' of geranylmethyl at the C-2 of 2*H*-pyran ring. The methyl group substitution at C-6 was inferred by HMBC interactions of H-7 (δ 6.49) to C-11 (δ 15.4, CH_3_). The complete ^1^H- and ^13^C-NMR chemical shift assignments, ^1^H–^1^H COSY and HMBC correlations, are presented in Table 1. The ^1^H- and ^13^C-NMR spectroscopic data of **1** were similar to those of 2-(4',8'-dimethylnona-3',7'-dienyl)-8-hydroxy-2-methyl-*2H*-chromene-6-carboxylic acid methyl ester [27], which was previously isolated from *Piper umbellatum* and *Piper peltatum*. The only exception is that the methyl ester at the C-6 position in 2-(4',8'-dimethylnona-3',7'-dienyl)-8-hydroxy-2-methyl-2*H*-chromene-6-carboxylic acid methyl ester was replaced by a methyl group. Additionally, acetylation of **1** yielded the monoacetylated derivative **6**, which displayed a [M + Na]^+^ at 391.2249 in HRESIMS, thus a molecular formula of C_24_H_32_O_3_ was proposed for **6**. Indeed, the resonance due to H-7 was observed at δ 6.68 (C-7/δ 122.8), showing the expected downfield shift in comparison to **1** and thus confirmed the location of acetyl group at C-8. The geometry of double bond C-3'/C-4' of geranylmethyl was assigned as *E* on the basis of the NOESY correlations of H_2_-2'/H_3_-11' and H-3'/H_2_-5'. Moreover, compound **1** did not show any significant Cotton effects and its poor optical activity indicates possibly it is a 1:1 mixture of two enantiomers [25]. Based on the above evidences and detailed analyses of NMR spectra, the structure of compound **1** was determined as 2-(4',8'-dimethylnona-3'*E*,7'-dienyl)-8-hydroxy-2,6-dimethyl-2*H*-chromene.

This chemical investigation also afforded four known compounds, methylfarnesylquinone (**2**) [8], isololiolide (**3**) [9], pheophytin a (**4**) [10,11], and β-carotene (**5**) [12]. Compounds **2**–**5** were identified by comparing their UV, IR and NMR spectroscopic data with those reported in the literature.

**Table 1:** ^1^H- and ^13^C-NMR data, ^1^H–^1^H COSY, and HMBC correlations of **1**.

Position	^1^H *^a^*	^13^C *^b^*	^1^H–^1^H COSY	HMBC
2		77.8 (C)		
3	5.60 d (9.6)	130.7 (CH) *^d^*	H-4	C-2, C-10
4	6.25 d (9.6) *^c^*	122.8 (CH)	H-3	C-2, C-5, C-8, C-10
5	6.34 d (2.8)	110.3 (CH)		C-4, C-7, C-8
6		126.3 (C)		
7	6.49 d (2.8)	117.1 (CH)		C-5, C-8, C-11
8		144.8 (C)		
9		148.5 (C)		
10		121.4 (C)		
11	2.15 s	15.4 (CH_3_)		C-6, C-7, C-8
12	1.38 s	25.8 (CH_3_)		C-2, C-3, C-1'
1'	1.72 m	40.7 (CH_2_)	H_2_-2'	C-2, C-2'
2'	2.12 m	22.5 (CH_2_)	H_2_-1', H-3'	
3'	5.14 (overlapped with H-7')	124.0 (CH)	H_2_-2'	C-2', C-5', C-11'
4'	135.2 (C)		
5'	1.98 m	39.6 (CH_2_)	H_2_-6'	C-4', C-6', C-7'
6'	2.06 m	26.6 (CH_2_)	H_2_-5', H-7'	C-4', C-5', C-7'
7'	5.11 (overlapped with H-3')	124.3 (CH)	H_2_-6'	C-6', C-9', C-10'
8'		131.3 (C)		
9'	1.70 s	25.6 (CH_3_)		C-7', C-10'
10'	1.61 s	17.6 (CH_3_)		C-7', C-8'
11'	1.60 s	15.8 (CH_3_)		C-4', C-5'
8-OH	5.35 s			

*^a^* Spectra recorded at 400 MHz in CDCl_3_ at 25 °C; *^b^* Spectra recorded at 100 MHz in CDCl_3_ at 25 °C; *^c^ J* values (in Hz)in parentheses; *^d^* Attached protons were deduced by DEPT experiments.

Compounds **1**, **2** and **4**–**6** were tested for cytotoxic effects against HepG2, A-549, and MDA-MB-231 cancer cell lines using the MTT assay. As shown in Table 2, compounds **1**, **2** and **6** revealed moderate to weak cytotoxicity with IC_50_ values ranging from 20.2 to 43.1 μM and the remaining compounds were inactive. These results revealed that hydroxy chromene (**1**) was more effective than its acetyl derivative (**6**). Compound **1** exhibited significant antibacterial activity (Table 3) against *S. marcescens* with a dosage of 10 μg/disk (Inhibition zone: 1.0 mm) when compared to that of standard antibiotic ampicillin with a dosage of 100 μg/disk (Inhibition zone: 1.5 mm). In addition, compounds **1**, **2**, **5** and **6** showed good potential against *P. aeruginosa* compared to that of ampicillin.

**Table 2:** Cytotoxicity data of compounds **1**, **2** and **4**–**6**.

Compounds	Cell Lines, IC_50_ (μM)		
	Hep G2	A549	MDA-MB-231
**1**	22.4	43.1	20.2
**2**	30.1	27.0	26.0
**4**	- ^a^	-	-
**5**	-	-	-
**6**	41.5	38.2	35.9
Doxorubicin ^b^	0.3	1.9	2.1

^a^ Inactive with IC_50_ > 50 μM; ^b^ Doxorubicin was used as a reference compound.

**Table 3:** Antibacterial activity (zone of inhibition in mm) of compounds **1**, **2** and **4**–**6**.

Compounds	Dosage (μg/disk)	Inhibition Zone (mm)					
		*B. cereus*	*S. aureus*	*S. typhimurium*	*P. aeruginosa*	*S. marcescens*	*Y. enterocolitica*
**1**	100				2.5		
	40	1.0	3.0	0.5		1.5	2.0
	30	0.5	1.0	0.5		1.5	1.5
	20	2.0	3.5	1.5		3.0	1.0
	15	1.5	2.5	1.5		2.5	2.0
	10	1.0	2.0	2.0		1.0	2.0
	5						1.0
**2**	60				1.0		
	50				0.5		
	30				0.5		
**4**	20						2.5
	15						1.0
	10						0.5
	5						0.5
**5**	30				0.5		
	20				0.5		
**6**	60			1.0			
	50			1.0			
	30			0.5			
	20				1.0		
Ampicillin	300	4.0	13.0	10.0	0.0	4.0	
	200	3.5	13.0	8.5	0.0	2.5	
	100	3.5	12.0	6.5	0.0	1.5	18.0
	50	3.0	10.5	5.0	0.0		11.0
	25		8.5	4.0	0.0		4.0

The *in vitro* anti-inflammatory activity of compounds **1**, **2**, **5**, and **6** were evaluated by suppressing *N*-formyl-methionyl-leucylphenylalanine/cytochalasin B (FMLP-CB)-induced superoxide anion generation and elastase release in human neutrophils (Table 4). Among them, compound **2** was found to display potent inhibitory effects on generation of superoxide anion (IC_50_ 0.22 ± 0.03 μg/mL) and the release of elastase (IC_50_ 0.48 ± 0.11 μg/mL) by human neutrophils at a concentration of 10 μg/mL.

**Table 4:** Inhibitory effects of compounds **1**, **2**, **5** and **6** on superoxide anion generation and elastase release in FMLP/CB-induced human neutrophils at 10 μg/mL.

Compounds	Superoxide Anion		Elastase Release	
	IC_50_ (μg/mL) ^a^	Inh %	IC_50_ (μg/mL) ^a^	Inh %
**1**		−16.34 ± 1.87 ***		−30.28 ± 1.49 ***
**2**	0.22 ± 0.03	91.63 ± 1.30 ***	0.48 ± 0.11	117.22 ± 6.53 ***
**5**	>10	38.42 ± 5.79 **	>10	43.48 ± 4.36 ***
**6**	>10	8.31 ± 3.25	>10	14.62 ± 3.84 *

Percentage of inhibition (Inh %) at 10 μM concentration. Results are presented as mean ± S.E.M. (the standard error of mean) (*n* = 3 or 4). * *p* < 0.05, ** *p* < 0.01, *** *p* < 0.001 compared with the control value. ^a^ Concentration necessary for 50% inhibition (IC_50_).

## 3. Experimental Section

### 3.1. General Experimental Procedures

Optical rotations were measured on a JASCO P1020 polarimeter (JASCO, Tokyo, Japan). IR spectra were recorded on a JASCO FT/IR-4100 spectrophotometer (JASCO, Tokyo, Japan). Ultraviolet spectra were recorded on a JASCO V-650 spectrophotometer (JASCO, Tokyo, Japan). The NMR spectra were recorded in CDCl_3_ on a Varian MR-400 FT-NMR spectrometer (Varian, Palo Alto, CA, USA). ESIMS and HRESIMS were recorded by ESI FT-MS on a Bruker APEX II mass spectrometer (Bruker, Bremen, Germany). Silica gel 60 (230–400 mesh, Merck, Darmstadt, Germany) was used for column chromatography. Pre-coated silica gel plates (Kieselgel 60 F_254_ 0.25 mm, Merck) were used for TLC analyses. High-performance liquid chromatography was performed on a Hitachi L-7100 HPLC apparatus (Hitachi, Tokyo, Japan) with a C-18 column (250 mm × 10 mm, 5 μm, Supelco, Bellefonte, PA, USA).

### 3.2. Alga Material

The brown alga *H. formosana* was collected by hand via scuba off the coast of San-Hsian-Tai, in July 2008, at a depth of 2–5 m, and stored in a freezer until extraction. A voucher specimen (2008-SHTBA-001) was deposited in the Department of Marine Biotechnology and Resources, National Sun Yat-sen University.

### 3.3. Extraction and Isolation

The frozen bodies of *H. formosana* (1.3 kg, wet weight) were minced and exhaustively extracted with acetone (10 × 1.5 L, each for 3 days) at room temperature. The acetone extract (10.3 g) was fractionated by open column chromatography on silica gel using *n*-hexane-EtOAc and EtOAc-MeOH mixtures of increasing polarity to yield 40 fractions. Fraction 4 was eluted with *n*-hexane-EtOAc (80:1) to afford **5** (12.1 mg). Fraction 9, eluted with *n*-hexane-EtOAc (30:1), was subjected to a Sephadex LH-20 column, using acetone as the mobile phase, to afford **2** (33.5 mg). Fraction 13, eluting with *n*-hexane-EtOAc (4:1), was subjected to a Sephadex LH-20 column, using acetone as mobile phase, to afford five separated subfractions. Subfraction 3 was further separated by silica gel column (*n*-hexane-Acetone, 10:1) to afford **1** (65.8 mg). Fraction 15, eluted with *n*-hexane-EtOAc (1:1), was subjected to a Sephadex LH-20 column, using acetone as mobile phase, to afford three separated subfractions. Subfraction 1 was further purified by silica gel column (*n*-hexane-dichloromethane-acetone, 10:1:1) to afford **4** (3.0 mg). Fraction 18, eluted with *n*-hexane-EtOAc (1:2), was subjected to a Sephadex LH-20 column, using acetone as mobile phase, to afford five separated subfractions. Subfraction 4 was further separated by silica gel column (*n*-hexane-dichloromethane-acetone, 10:1:1) to afford **3** (1.5 mg).

2-(4',8'-Dimethylnona-3'*E*,7'-dienyl)-8-hydroxy-2,6-dimethyl-2*H*-chromene (**1**): Pale yellow oil; ${\left[\text{α}\right]}_{\text{D}}^{25}$ = +0.5 (*c* 0.25, CHCl_3_); UV (MeOH) λ_max_ (log ε) 206 (4.4), 231 (4.3), 265 (3.6), 274 (3.6), 333 (3.4) nm; IR (KBr) *v*_max_ 3396, 2922, 1729, 1593, 1463, 1311, 853, and 717cm^−1^; ^13^C- and ^1^H-NMR data, see Table 1; ESIMS *m*/*z* 349 [M + Na]^+^; HRESIMS *m*/*z* 349.2146 [M + Na]^+^ (calcd. for C_22_H_30_O_2_Na, 349.2143).

### 3.4. Acetylation of Compound **1**

Compound **1** (5.0 mg) was dissolved in pyridine (0.2 mL) and then Ac_2_O (0.1 mL) was added. After 24 h at room temperature, the reaction residue was subjected to column chromatography over silica gel using *n*-hexane-EtOAc (5:1) to yield the acetyl derivative **6** (4.1 mg).

2-(4',8'-Dimethylnona-3'*E*,7'-dienyl)-8-acetyl-2,6-dimethyl-2*H*-chromene (**6**): Colorless oil; ${\left[\text{α}\right]}_{\text{D}}^{25}$ = +0.3 (*c* 0.1, CHCl_3_); UV (MeOH) λ_max_ (log ε) 205 (4.5), 227 (4.4), 266 (3.6), 275 (3.5), 318 (3.4) nm; IR (neat) ν_max_ 2922,1760, 1468, 1369, 1207, 1020, 894, and 723 cm^−1^; ^1^H-NMR (400 MHz, CDCl_3_): δ 5.58 (1H, d, *J* = 9.6 Hz, H-3), 6.27 (1H, d, *J* = 9.6 Hz, H-4), 6.68 (1H, d, *J* = 2.8 Hz, H-7), 6.55 (1H, d, *J* = 2.8 Hz, H-5), 2.16 (3H, s, 6-CH_3_), 1.38 (3H, s, 2-CH_3_), 2.16 (3H, s, 8-OAc), 1.71 (2H, m, H-1'), 2.11 (2H, m, H-2'), 5.14 (1H, overlapped, H-3'), 1.97 (2H, m, H-5'), 2.05 (2H, m, H-6'), 5.11 (1H, overlapped, H-7'); ^13^C-NMR (100 MHz, CDCl_3_): δ 78.5 (C-2), 130.3 (C-3), 122.5 (C-4), 116.5 (C-5), 126.3 (C-6), 122.8 (C-7), 148.7 (C-8), 143.3 (C-9), 120.9 (C-10), 26.5 (C-3/CH_3_), 15.5 (C-6/CH_3_), 21.1 (C-8/OAc), 41.2 (C-1'), 22.6 (C-2'), 123.9 (C-3'), 135.3 (C-4'), 39.7 (C-5'), 26.7 (C-6'), 124.3 (C-7'), 131.3 (C-8'), 15.9 (C-4'/CH_3_), 17.7 (C-8'/CH_3_), 25.7 (C-8'/CH_3_); ESIMS *m*/*z* (relative intensity) 391 [M + Na]^+^; HRESIMS *m*/*z* 391.2249 [M + Na]^+^ (calcd. for C_24_H_32_O_3_Na, 391.2246).

### 3.5. Cytotoxicity Testing

Cell lines were purchased from the American Type Culture Collection (ATCC). Cytotoxicity assays of compounds **1**, **2** and **4**–**6** were performed using the MTT [3-(4,5-dimethylthiazol-2-yl)-2,5-diphenyl-tetrazolium bromide] colorimetric method [28,29].

### 3.6. In Vitro Antibacterial Assay

The antibacterial activity of the isolated compounds was evaluated against *Bacillus cereus* (ATCC14579), *Staphylococcus aureus* (ATCC9144), *Salmonella typhimurium* (ATCC914028), *Pseudomonas aeruginosa* (ATCC27853), *Serratia marcescens* (ATCC25419), and *Yersinia enterocolitica* (ATCC23715), based on previous reports [30,31]. Bacterial strains were grown in LB (Luria-Bertani) broth medium for 24 h at 37 °C. Then, 17 mL LB hard agar (1.5% agar) was poured into sterile Petri dishes (90 mm) and allowed to set. When testing the bacterial sample, 1000 μL of inoculum suspension was poured into the molten LB soft agar plates using a sterile micropipet. After the temperature reached around 55–60 °C, and the mixture was homogenized thoroughly by mixing in a circular motion (pour-plate technique). Sterile paper disks (Advantec, 8 mm in diameter) were placed onto the top layer of the LB agar plates. Test compounds (2 μg/μL) of different volumes were applied on each of the filter paper disks. Ampicillin (5 μg/μL) with different volumes of various dosages and DMSO were served as positive and negative controls respectively. All the plates were incubated at 37 °C, 24 h prior to the evaluation of antibacterial activity.

### 3.7. In Vitro Anti-Inflammatory Assay—Superoxide Anion Generation and Elastase Release by Human Neutrophils

Human neutrophils were obtained by means of dextran sedimentation and Ficoll centrifugation. Measurements of superoxide anion generation and elastase release were carried out according to previously described procedures [32,33]. Briefly, superoxide anion production was assayed by monitoring the superoxide dismutase-inhibitable reduction of ferricytochrome c. Elastase release experiments were performed using MeO-Suc-Ala-Ala-Pro-Val-*p*-nitroanilide as the elastase substrate [34].

## 4. Conclusions

A new chromene derivative 2-(4',8'-dimethylnona-3'*E*,7'-dienyl)-8-hydroxy-2,6-dimethyl-2*H*-chromene (**1**) together with four known natural products **2**–**5** were isolated from the brown alga *H. formosana*. Both compounds **1** and **2** displayed marginal cytotoxicities toward HepG2, A-549, and MDA-MB-231 cancer cell lines. Compounds **1**, **2**, **5** and **6** showed potent inhibition against *P. aeruginosa* compared to that of ampicillin. Methylfarnesylquinone **2** revealed potent *in vitro* anti-inflammatory activity by inhibiting the generation of superoxide anion (IC_50_ 0.22 ± 0.03 μg/mL) and the release of elastase (IC_50_ 0.48 ± 0.11 μg/mL) in FMLP/CB-induced human neutrophils. Thus, compound **2** can be considered as a candidate for future anti-inflammatory drug development.

## Supplementary Materials

Supplementary figures can be found at: http://www.mdpi.com/1422-0067/16/01/0736/s1.
